# Health Care Providers’ Experiences and Perceptions With Telehealth Tools in a Hospital-at-Home Program: Mixed Methods Study

**DOI:** 10.2196/56860

**Published:** 2025-04-17

**Authors:** Shi Yun Low, Stephanie Qianwen Ko, Ian Yi Han Ang

**Affiliations:** 1 NUHS@Home, National University Health System, Singapore, Singapore; 2 Division of Advanced Internal Medicine, Department of Medicine, National University Hospital, 1E Kent Ridge Road, Singapore, 119228, Singapore, 65 9879 5566; 3 Saw Swee Hock School of Public Health, National University of Singapore, Singapore, Singapore

**Keywords:** telehealth usability, hospital-at-home, health care provider experience, virtual consultation, vital signs monitoring, mixed-methods study, health care provider, experience, perception, telehealth tools, telehealth, e-consultations, teleconsultation, hospital-based, home-based, mobile phone

## Abstract

**Background:**

The growing demand for hospital-based care, driven by aging populations and constrained resources, has accelerated the adoption of telehealth tools such as teleconsultations and remote monitoring in hospital-at-home (HaH) programs. Despite their increasing use in delivering acute care at home, studies exploring health care providers’ experiences and perceptions of these tools within HaH settings remain limited.

**Objective:**

This study aimed to understand the experiences and perspectives of health care providers toward teleconsultations and vital signs monitoring systems within a HaH program in Singapore to optimize effectiveness and address challenges in future implementation.

**Methods:**

A convergent mixed methods approach that combines qualitative in-depth interviews with an electronic survey designed based on the 5 domains (usefulness, ease of use, effectiveness, reliability, and satisfaction) of the Telehealth Usability Questionnaire was used.

**Results:**

In total, 37 surveys and 20 interviews were completed. Participants responded positively to the use of both teleconsultation and vital signs monitoring with a mean total score of each method being 4.55 (SD 0.44) and 4.52 (SD 0.42), respectively. Significantly higher mean ratings were observed among doctors compared with other health care providers for usefulness (*P*=.03) and ease of use (*P*=.047) in teleconsultations. Health care providers with fewer years of clinical experience also perceived the use of vital signs monitoring to be more effective (*P*=.02) and more usable (*P*=.04) than those with more years of experience. Qualitative analysis identified four themes: (1) benefits of telehealth for health care providers such as improved work convenience, efficiency, and satisfaction; (2) challenges of telehealth implementation relating to communication and technology; (3) perspectives on telehealth impact; and (4) enablers for successful implementation. Comparing both datasets, qualitative findings were aligned with and confirmed quantitative results.

**Conclusion:**

This study highlighted the benefits and usability of telehealth among health care providers. However, challenges relating to patient communication, technological issues, and delivery of care were also discussed along with enablers for successful implementation. These insights can inform strategies to optimize future implementation of telehealth in HaH.

## Introduction

As health care systems worldwide face growing challenges from aging populations, the demand for hospital-based care continues to rise, putting increasing pressure on limited health care resources [1,2]. In response, innovative care models such as hospital-at-home (HaH) have been developed to meet these demands [3]. Although the concept of HaH is not novel, its adoption has gained momentum in recent years driven by advancements in digital health technologies, with successful implementations in the United States, Europe, Australia, and Singapore [4,5]. By leveraging these technologies, HaH aims to optimize health care delivery, allowing more patients who require hospital admission to receive treatment at home and supporting the early discharge of patients whose care can safely continue outside the hospital [6,7]. Likewise, HaH played a critical role in supporting health care systems during the COVID‐19 pandemic [8-11], drawing attention to its potential as a promising care model that extends coverage for pandemic and nonpandemic times.

The integration of digital health tools, such as remote monitoring systems and telecommunication technologies in HaH, allow health care providers to remotely assess patients via video or telephone consultations and monitor self-reported vital signs and symptoms, creating a seamless and efficient care experience [10-12]. Studies have consistently shown that these approaches are both safe and effective, with low mortality and return to hospital admissions rates [10,11,13,14], and high patient satisfaction levels, as patients feel reassured knowing their conditions are remotely monitored [13-17].

Despite these promising outcomes, there remains a lack of research exploring the experiences and perspectives of health care providers using telehealth and remote monitoring tools in the HaH setting. Existing studies that have explored health care provider experiences with telehealth use have primarily focused on routine follow-up in outpatient clinics [18-20], long-term care [21,22], and palliative setting [23] rather than in the HaH setting caring for acutely ill patients. Understanding these experiences can help design appropriate training for health care workers looking to work in HaH settings and develop appropriate support systems to ensure health care workers’ well-being, the fourth domain of the Quadruple Aim [24] .

To address this gap, we aimed to understand the experiences and perspectives of health care providers toward teleconsultations and vital signs monitoring systems within an HaH program.

## Methods

### Study Design

This study adopted a convergent mixed methods design. Quantitative data from an electronic telehealth usability survey and qualitative data from in-depth interviews were collected simultaneously during a single data collection phase. These datasets were then analyzed separately before being interpreted collectively [25]. The mixed methods approach was chosen for its capacity to enhance data interpretation by leveraging the complementary strengths of each method. The quantitative survey data offer statistical evidence and a general overview of health care providers’ perceptions regarding the usability of telehealth in HaH. On the other hand, qualitative interviews provide valuable contextual information and deeper insights. By integrating both datasets, a more comprehensive understanding of health care providers’ experiences and perspectives can be achieved [26].

### Setting and Sample Frame

The National University Health System (NUHS) is one of Singapore’s 3 public health care clusters, responsible for taking care of the Western population of Singapore. The HaH program at NUHS, known as NUHS@Home, offers home-based hospitalization services to 3 acute hospitals within the health care cluster [4]. In September 2021, NUHS@Home expanded its services to include patients with COVID-19 to manage the surge in cases. To accommodate the larger volume of patients, patients’ daily clinician review transitioned from in-person home visits to teleconsultations (both video and audio) with the care team that consists of doctors, nurses, pharmacists, and allied health professionals.

Patients admitted in NUHS@Home during the study period were primarily older adults with COVID-19 infection. Most were proficient in smartphone use or have younger caregivers comfortable with using technology to manage patient care at home. Patients received daily regular scheduled teleconsultations using their own mobile devices and reported their home-monitored vitals through a chatbot with an online reporting form. For patients less proficient with technology, automated vital sign monitoring kits were provided, which transmitted home-monitored vitals to the care team and included a tablet for teleconsultations. Instructions on remote vital signs monitoring and frequency of teleconsultations were provided to patients or their caregivers before their admission to NUHS@Home.

Purposive sampling was used to select participants for this study. All health care providers who were involved in delivering care within NUHS@Home between September 2021 and December 2022 were sampled. Those that have used teleconsultation, vital signs monitoring, or both telehealth methods to provide care in NUHS@Home, were eligible for the study.

### Data Collection

#### Overview

Participants were recruited via an email invitation sent out by the primary investigator of the study (SQK) between November 2022 and January 2023. Recruitment for the study concluded when no new survey responses were received, and data saturation was achieved for the qualitative component of the study.

#### Surveys

We chose to adopt the Telehealth Usability Questionnaire (TUQ), which assesses usability across 5 domains, that are usefulness, ease of use, effectiveness, reliability, and satisfaction [27]. The TUQ was selected as it has been widely used to capture user perspectives on telehealth utilization [28]. It has demonstrated strong content validity and internal consistency, with Cronbach α values ranging from 0.81 to 0.93 for each domain [27]. The electronic survey included 21 TUQ questions specific to teleconsultation and the same set of 21 questions related to vital signs monitoring. Demographic information of health care providers, their roles in NUHS@Home, previous telehealth experience, and years of clinical experience were also collected. Survey questions were presented in English, and responses to TUQ questions were rated on a 5-point Likert scale (ranging from strongly disagree [−1] to strongly agree [−5]).

#### In-Depth Interviews

Participants who completed the electronic survey were also invited to participate in an additional in-depth interview. A semistructured interview guide was developed based on the TUQ domains and existing literature on health care providers’ telehealth experiences [29-32]. The interview guide was pilot tested with 2 other health care providers before the actual interviews. All interviews were conducted online in English via Zoom (Zoom Communications, Inc) video call and audio recorded for analysis. Each interview lasted approximately between 30 and 90 minutes. The interviews were conducted and transcribed by the first author of this paper (SYL), a Master of Public Health graduate student with qualitative training. Given the comprehensive nature of the interview data collected, no repeat interviews were conducted, and transcripts were not returned to participants for clarification.

### Data Analysis

Survey data, including participant characteristics and TUQ scores, were analyzed and summarized using descriptive statistics. Percentages of responses for each Likert scale point were calculated for each question. The TUQ total score was obtained by calculating the mean of responses for all 21 questions. TUQ subscale scores were calculated with the mean of the responses for the questions in each of the 5 domains. Means and SDs of TUQ total and subscale scores are presented. As the TUQ total and subscale scores did not follow a normal distribution, the Mann-Whitney *U* test was used to examine differences in scores between professional groups, years of clinical experience, and previous telehealth experience. The significance level was set at α=.05. Statistical analysis was conducted using the R statistical software version 4.2.2 (R Core Team).

All audio-recorded interviews were transcribed verbatim, excluding any local Singaporean slang, and participant identifiers were omitted during the transcription. The transcripts were analyzed using an inductive reflexive thematic approach outlined by Braun and Clarke [33] and Byrne [34]. Transcripts were coded line-by-line using ATLAS.ti Web software v5.16.1-2023-10-14 (Lumivero) by SYL with no second coder, prioritizing the researcher’s reflexive and thoughtful engagement with both the data and the analytic process. To ensure the rigor and trustworthiness of emerging subthemes and themes, the meanings of codes were compared and discussed monthly among all authors of this study in an iterative and collaborative process.

Upon completing analysis for both sets of data, qualitative findings were compared with quantitative results by TUQ domains to assess confirmation, expansion, or discordance between the datasets and to draw meta-inferences [35]. Data integration was completed by SYL, and the Good Reporting of a Mixed Methods Study criteria [36] guided the reporting of results to ensure reporting quality (Multimedia Appendix 1).

### Ethical Considerations

This study was approved by the National Healthcare Group Domain Specific Review Board (Ref 2021/00896). Since the survey was anonymous, the requirement for informed consent from survey participants was waived. To maintain anonymity for the survey, respondents who were interested in participating in the interviews were asked to voluntarily provide their contact information in a separate, optional section of the survey. This ensured that survey responses remained anonymous and unlinked to identifying information unless the respondent explicitly chose to participate in the interview. Informed consent was only obtained from participants who expressed interest in participating in the in-depth interviews. All data collected and analyzed were anonymized. Each interviewed participant was also compensated for their time and involvement with SGD $20 (SGD $1=US $0.75).

## Results

### Participant Demographics

A total of 49 health care providers were invited to participate in the study. Among the 49 eligible health care providers, 37 responded and completed the electronic survey, resulting in a response rate of 76%. Out of the 37 survey participants, 20 also took part in the interviews. While 59% (22/37) of the participants were doctors, the remaining participants represented a diverse mix of health care providers of various professions such as nurses, pharmacists, and allied health professionals (Table 1). In total, 76% (28/37) of participants reported using both teleconsultation and vital signs monitoring in NUHS@Home. The participants had a median of 7 (IQR 4‐10) years of clinical experience, but 35% (13/37) of them did not have any previous experience with telehealth.

**Table 1.** Characteristics of participants.

Characteristics		Survey participants (n=37)	Interview participants (n=20)
Age group (years), n (%)			
	20‐29	9 (24)	4 (20)
	30‐39	22 (59)	13 (65)
	40‐49	5 (14)	2 (10)
	50‐59	1 (3)	1 (5)
Sex, n (%)			
	Male	15 (41)	9 (45)
	Female	22 (59)	11 (55)
Ethnicity, n (%)			
	Chinese	33 (89)	18 (90)
	Malay	1 (3)	1 (5)
	Indian	1 (3)	0 (0)
	Other	2 (5)	1 (5)
Profession, n (%)			
	Doctor	22 (59)	9 (45)
	Nurse	5 (14)	4 (20)
	Allied health professional	7 (19)	5 (25)
	Pharmacist	3 (8)	2 (10)
Telehealth methods used, n (%)			
	Teleconsultation	3 (8)	2 (10)
	Vital signs monitoring	6 (16)	5 (25)
	Both methods	28 (76)	13 (65)
Days per week involved in NUHS@Home, median (IQR)		4 (2-5)	4 (2-5)
Years of clinical experience, median (IQR)		7 (4-10)	8 (5-13)
Previous experience with telehealth, n (%)			
	No experience	13 (35)	6 (30)
	<3 months	4 (11)	3 (15)
	3‐6 months	4 (11)	1 (5)
	6‐9 months	5 (14)	2 (10)
	9‐12 months	3 (8)	0 (0)
	>12 months	8 (22)	8 (40)

### Survey Results

**Figure 1.**
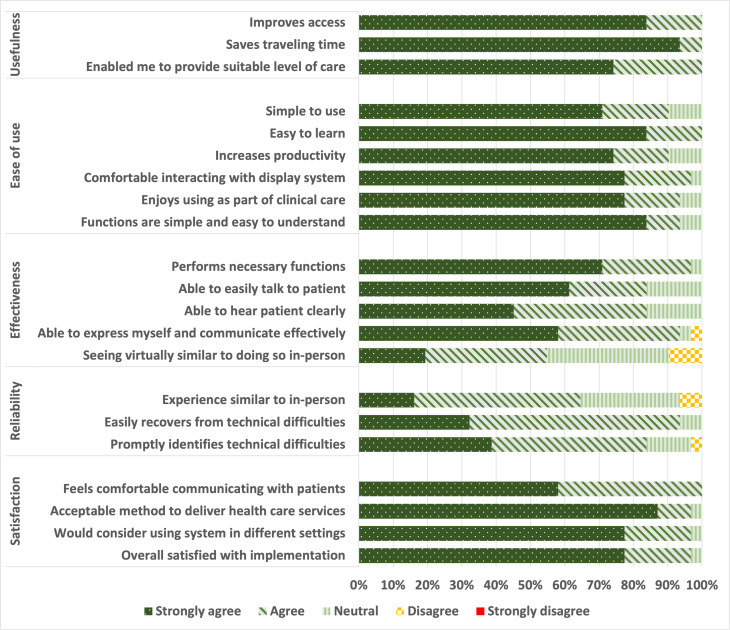
Telehealth Usability Questionnaire responses for teleconsultation.

**Figure 2.**
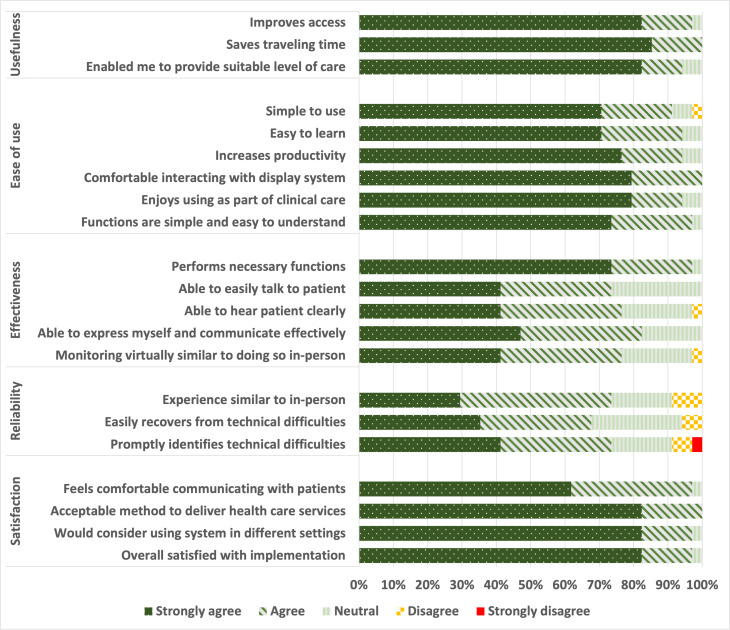
Telehealth Usability Questionnaire responses for vital signs monitoring.

In total, 31 responses were collected for the use of teleconsultation in NUHS@Home. The TUQ total score for teleconsultation ranged from 3.4 to 5, with a mean score of 4.55 (SD 0.44). For teleconsultation, the mean TUQ subscale scores for each domain, from highest to lowest scores, were as follows: “usefulness” (mean 4.84, SD 0.30), “satisfaction” (mean 4.73, SD 0.43), “ease of use” (mean 4.72, SD 0.49), “effectiveness” (mean 4.31, SD 0.61), and “reliability” (mean 4.06, SD 0.57) (Figure 1). Furthermore, 34 responses were collected for vital signs monitoring. The TUQ total scores for vital signs monitoring ranged from 3.5 to 5, with a mean score of 4.52 (SD 0.42). For vital signs monitoring, the TUQ subscale scores for “usefulness” (mean 4.8, SD 0.36), “satisfaction” (mean 4.75, SD 0.35), and “ease of use” (mean 4.7, SD 0.43) were high (Figure 2). However, “effectiveness” (mean 4.29, SD 0.59) and “reliability” (mean 3.98, SD 0.87) scored slightly lower. Further breakdown of TUQ scores is available in Multimedia Appendix 2.

Doctors perceived teleconsultation to be more useful compared with other health care providers (mean usefulness 4.91 vs 4.67; *P*=.03). Similarly, doctors found teleconsultation easier to use compared with other health care providers (mean ease of use 4.87 vs 4.35; *P*=.047). No significant differences in TUQ subscale scores between doctors and other health care providers were observed for other TUQ domains of teleconsultation or vital signs monitoring. Health care providers with fewer years of clinical experience perceived the use of vital signs monitoring to be more effective (mean effectiveness 4.48 vs 4.04; *P*=.02) and more usable (mean 4.63 vs 4.39; *P=*.04) than those with more years of clinical experience. No significant differences in TUQ scores were observed for teleconsultation or other TUQ domains of vital signs monitoring based on years of clinical experience. Furthermore, no significant differences in TUQ scores were found when comparing health care providers based on previous telehealth experience for both teleconsultation and vital signs monitoring (Multimedia Appendix 3).

### Interview Results

Thematic analysis of the interview transcripts yielded 62 unique codes, which were grouped into 4 overarching themes and 8 subthemes. The 4 themes identified are benefits of telehealth tools for health care providers (23 codes and 135 quotes), challenges of telehealth implementation (16 codes and 133 quotes), perspectives on telehealth impact (14 codes and 84 quotes), and enablers for successful implementation (9 codes and 66 quotes). A summary of themes, subthemes, and examples of codes and participant quotes is presented in Table 2.

**Table 2.** Summary of participants’ themes, subthemes, codes, quotes.

Themes, subthemes, and codes			Examples of quotes
Benefits of telehealth for healthcare providers			
	Improved work convenience, efficiency, and productivity		
		Easy to learn and use vital signs monitoring systems	“…it’s a very straightforward system that is very easy to learn to navigate, very easy to use.” [ID9, male doctor]
		Reduced need for traveling	“So I think teleconsult has it’s good because we actually cut down traveling and received a lot of time in return. The patient is just a phone call or a video call away… You just have to have a set of working equipment in front of you, and you can just sit in front of that and provide care for your patients.” [ID15, female nurse]
		Ability to use telehealth methods to effectively identify signs of deterioration	“…through the patients submitting their vitals, actually see and catch any red flags immediately. You know, like persistent high blood pressure, then we know there’s something going on. So we can provide treatment immediately.” [ID1, female allied health professional]
	Enhanced work satisfaction and confidence		
		Improved teamwork and collaboration	“…because it’s all tele, so the team can be physically close together. That means the doctors, the nurses, the pharmacists, all in like one room… I think it’s good for like collaboration or when they have issues or when we have questions, it’s much easier to just reach them…” [ID5, male pharmacist]
		Facilitated physical distancing and protecting care providers against infectious diseases	“We don’t need to gown up and wear all the PPE. Yeah, so in terms of the safety of the staff and with infection control, this is definitely a plus point.” [ID20, female pharmacist]
		Improved care provider’s confidence in using telehealth	“…I come from a time where physical consultation is extremely important… So at the start, I don’t think my confidence was very high to do telemedicine. But after going through [HaH], the interactions with patients have been mostly pleasant… I think it has increased my competence and confidence in managing patients at home using telemedicine.” [ID13, male doctor]
Challenges of telehealth implementation			
	Difficulties in patient communication and engagement		
		Difficulty assessing nonverbal information and conducting physical assessments	“You don’t get to physically examine your patients. On top of that, because you are not seeing them fully, you can’t read their body language, you can’t assess a lot of things. So you end up having to talk a lot more to glean more information from a patient to make a clinical decision, as compared to in a ward or in a clinic where you can size up patient pretty fast.” [ID9, male doctor]
		Difficulty conveying information to patients	“I guess it’s a lot of repetition. Sometimes they don’t understand, or they can’t hear clearly over video [call]…. Or they can’t really operate the video calls very well.” [ID11, female doctor]
		Difficulty contacting noncompliant patients and patients at certain time of the day	“You get a few [patients] who just don’t want to comply with the rules like they don’t want to submit their vitals, they don’t want to turn on video because they’re sleeping, or they don’t want to pick up their phone call.” [ID17, female doctor]
	Technological inconveniences related to telehealth systems		
		Care providers encountering technical issues with vital signs monitoring systems	“Audio call, sometimes… including the video calls as well, sometimes it can be quite choppy. We then need to make the calls multiple times. So it tends to get a bit frustrating also.” [ID7, female nurse]
		Slow and tedious process to recover and manage technical issues	“Actually, we have feedback to the [vital signs monitoring system vendors] team. But somehow the team also can’t quite troubleshoot it when there’s problem.” [ID15, female nurse]
		Inconveniences encountered due to lack of interoperability of information technology platforms	“…you need to ensure you enter the patient’s details correctly. Because [vital signs monitoring system] itself, the patient details is not like an auto flow from [electronic medical records]. So if you enter wrongly or when you create the entry wrongly, then the data error sort of carries forward.” [ID3, male doctor]
	Operational issues		
		Increased risk of compromising patient privacy during virtual consults or unconducive environment to conduct virtual calls	“…it got a bit noisy, which is not that good and also can potentially compromise confidentiality because the guy next to you might be talking very loudly, asking for the NRIC number of his or her patients, and your patient might potentially hear the whole conversation.” [ID16, male doctor]
		Labor intensive for care providers to set up automated vital signs monitoring system	“The use of the automated kind of system where the vitals can automatically transmit into the system… involves a bit more resource setup, because someone needs to go to the patient’s house and set up the system there.” [ID14, male doctor]
Perspectives on telehealth impact			
	Perceived patient benefits		
		Perceived improved patient experience due to increased convenience and comfort	“…a shorter hospital stay, convalescing in a very comfortable home environment. So in terms of the experience for [the patients], it’s a lot better.” [ID12, male doctor]
		Improves patient empowerment	“[Patients] will have more self-awareness of their health. I think they will be more in control of their health when they monitor the blood pressure themselves.” [ID6, female nurse]
	Potential benefits to health care systems		
		Reducing need for hospital admissions and saving hospital resources	“…during COVID, one of the big issues was a lack of beds. So [telehealth] definitely helps to free up a lot of beds, especially for patients who are technically stable, who didn’t really need to be in [the hospital].” [ID17, female doctor]
		Increased capacity to care for more patients	“We can manage a large group of patients at once. We don’t need to visit all of them physically.” [ID10, male doctor]
	Potential risks and challenges		
		Concerns with accuracy of patient vitals submission	“Technically, it’s very difficult to find out if someone is actually, you know like faking the vitals reading… I think the readings that are taken at home will have some degree of lower reliability compared to in the hospital because the operators are the patients themselves and the caregivers rather than the nurses or the healthcare assistants.” [ID14, male doctor]
		Concerns of “overmonitoring”	“So we get patients who are very tired of being chased after to measure [vital signs] three times a day because they wouldn’t have otherwise be required to monitor their health at all… Also because of the timing that we would like to see the vital signs, we also ask them to do it at 7am, which is not their usual wake up time.” [ID7, female nurse]
Enablers to successful implementation			
		Suitability of using telehealth to manage patients with COVID-19	“…for COVID in particular, majority of the patients are fairly stable… there’s no reason to believe that they might deteriorate in front of your eyes. And I honestly tell you, medicine and monitoring are probably the preferred and sensible route… So honestly patients just need to go home…and can be monitored at home safely.” [ID12, male doctor]
		Importance of care provider-patient trust	“I think trust is implicit, we just have to trust that the numbers they are submitting is truly what they’re doing.” [ID13, male doctor]

### Integrating Survey and Interview Results

Quantitative and qualitative findings were summarized and meta-inferences were drawn in Table 3. Overall, high levels of usefulness were attributed to perceptions of increased convenience, efficiency, and usefulness. High satisfaction rates were attributed to a positive work environment, teamwork and collaboration, protection from infectious diseases, and high levels of acceptance described by participants. Systems were generally found to be easy to learn and use; however, lack of interoperability with electronic health records and noncompliant patients were cited as key challenges. The comparatively lower scores for effectiveness were likely associated with challenges relating to patient communication and engagement. Finally, the lower scores in the “reliability” domain were associated with technical inconveniences, such as connectivity issues and having a lengthy turnaround time to resolve technical issues.

**Table 3.** Integration of findings and the mixed methods meta-inference.

TUQ^a^ domain	Quantitative results	Qualitative results	Meta-inference
Usefulness	Mean TUQ subscale scores of 4.84 and 4.8 for the use of both teleconsultation and vital signs monitoring respectively.100% and 98% of participants rated positively^b^ to use of virtual consultation and vital signs monitoring, respectively.	Benefits of telehealth: Improved convenience due to reduced need for traveling Increased flexibility and ease of reaching out to their patients Efficient identification of early signs of deterioration Perceptions: Perceived patient benefits (eg, patient empowerment, patient comfort, and convenience) Potential benefits to health care system (eg, saving hospital resources and increased capacity to care for more patients) Enablers: Suitability of using telehealth to care for patients with COVID-19	Confirmation: participants’ positive experiences relating to improved convenience and efficiency, as well as their perception of the suitability of using telehealth to provide care for patients with COVID-19, align with the high scores observed in the “usefulness” domain. Expansion: participants also shared on their perceived benefits of using telehealth to both patients and the health care system, thus expanding on the concept of usefulness of telehealth not just for health care providers but also for patients and health care system.
Satisfaction	Mean TUQ subscale scores of 4.73 and 4.75 for the use of both teleconsultation and vital signs monitoring, respectively.98% and 97% of participants rated positively^b^ to use of virtual consultation and vital signs monitoring, respectively.	Benefits of telehealth: More opportunity for collaboration and sharing of expertise Improvement in work conditions Enjoyable virtual interactions Increased confidence and acceptance of telehealth	Confirmation: improvements in work conditions, protection from infectious diseases, improved teamwork and collaboration, and increased acceptance of telehealth described by participants were able to explain and confirm the relatively high scores rated for the “satisfaction” domain.
Ease of Use	Mean TUQ subscale scores of 4.72 and 4.70 for the use of both teleconsultation and vital signs monitoring, respectively.94% and 95% of participants rated positively^b^ to use of virtual consultation and vital signs monitoring, respectively.	Benefits of telehealth: Easy to learn and use vital signs monitoring systems Challenges of telehealth: Difficulty dealing noncompliant patients Inconveniences due to lack of interoperability between systems Labor intensive to set up automated systems Enablers: Using familiar platforms for virtual consultation	Confirmation: the majority of participants reported that the vital signs monitoring systems were easy to learn and use, with the utilization of familiar platforms for teleconsultations as an enabler. These findings corroborate the high scores observed in the “ease of use” domain. Expansion: a handful of the negative experiences encountered by the participants relating to inconveniences caused by the lack of interoperability between systems and setting up of automated systems, and difficulty dealing with noncompliant patients, could explain the lower percentage of participants who rated positively for the “ease of use” domain in telehealth use.
Effectiveness	Mean TUQ sub-scale scores of 4.31 and 4.29 for the use of both virtual consultation and vital signs monitoring, respectively.83% and 81% of participants rated positively^b^ to use of teleconsultation and vital signs monitoring, respectively.	Challenges of telehealth: Inability to assess nonverbal information Difficulty communicating with older adult patients Difficulty engaging and establishing rapport Unconducive environment Perceptions: Accuracy of patient vital signs submissions Fear of making wrong clinical decisions	Confirmation: the negative experiences reported by participants, specifically regarding challenges in patient communication and engagement, align with, and provide an explanation for the lower scores observed in the “effectiveness” domain.
Reliability	Mean TUQ subscale scores of 4.06 and 3.98 for the use of both teleconsultation and vital signs monitoring, respectively.81% and 72% of participants rated positively^b^ to use of teleconsultation and vital signs monitoring, respectively.	Challenges of telehealth: Technical issues with vital signs monitoring system Connectivity issues during virtual consultations Process of resolving such issues was often slow and cumbersome	Confirmation: recounts of negative experiences associated with the technical inconveniences related to telehealth systems, align with and confirm the lower scores observed in the “reliability” domain.

a Telehealth Usability Questionnaire.

b Rating either ”strongly agree” or ”agree.”

## Discussion

### Principal Findings and Comparison With Previous Work

In this mixed methods study exploring health care providers’ engagement in teleconsultations and vital signs monitoring for acute care at home, “usefulness,” “satisfaction,” and “ease of use” received the highest ratings, while “effectiveness” and “reliability” received lower ratings. These findings were corroborated during interviews, where overall positive perceptions were highlighted alongside key challenges related to patient communication and engagement, and technological issues.

Our study contributes both fresh insights and confirmation of existing literature on health care providers’ experiences with telehealth. Consistent with previous research [37], participants emphasized the convenience and flexibility of teleconsultations and vital signs monitoring, particularly in optimizing health care resources during the COVID-19 pandemic. This marks a shift from previous reports favoring telehealth use for chronic diseases [38] or palliative care [39] to acute care settings, possibly due to the unique demands of the pandemic. Challenges of managing noncompliant patients in HaH setting differed from stable chronic conditions in outpatient virtual settings, due to the acuity and risk of deterioration of these patients [40]. Concerns regarding vital signs accuracy and making clinical judgments using telehealth tools were also novel observations, possibly attributed to the adjustment from face-to-face consultations to telehealth adoption, especially among more experienced health care providers. Another noteworthy finding is that telehealth use in a multidisciplinary HaH setting brought health care providers closer together, fostering collaboration and shared learning opportunities.

On the other hand, participants who stressed the importance of interoperability between telehealth systems and electronic medical records were aligned with existing literature advocating for seamless integration and data exchange for successful telehealth implementation [41]. Reports of communication difficulties and patient engagement issues also mirror previous findings, underscoring the need for specific communication skills development among health care providers [42]. In addition, our study reaffirmed the impact of technological issues on telehealth reliability [43], emphasizing the need for robust technical support to ensure system stability, though participants mainly struggled with the slow resolution of problems rather than frequent connectivity or technical difficulties. Finally, participants’ high satisfaction with telehealth use echoes previous research [37,39,44], highlighting its acceptance and benefits across diverse care settings.

Health care providers’ real-world experiences offer vital insights that can help policy makers understand practical implications and identify policy gaps. Considerations for the real-world implementation and context can aid in crafting practical telehealth and HaH guidelines that reflect the realities of these settings. Health care systems can also learn from these experiences to create adaptable telehealth-ready systems to meet the needs of an aging population and improve health care accessibility, efficiency, and quality. Based on our findings, we recommend 3 key actions. First, strengthen health care provider training in developing skills for telecommunication, remote assessment, and decision-making, along with early exposure to digital health care tools. Second, ensure the reliability of telehealth systems through regular checks, updates, and robust technical support, and additionally integrate systems with electronic medical records to ensure efficient data exchange. Finally, promote multidisciplinary collaboration by designing physical spaces that facilitate effective communication and allocate private areas for teleconsultations to ensure patient confidentiality.

### Limitations

Our study has several limitations. First, the health care providers sampled were from a single HaH program, and their perspectives may not fully represent those of providers in other programs that use different telehealth tools or operate in varying contexts. Nonetheless, we included a diverse range of health care professions in both the survey and interviews to ensure a broad representation of experiences.

Second, the sample size in this study may be considered small, potentially limiting the statistical power of the quantitative analysis. However, the integration of qualitative data through a mixed methods approach mitigates this limitation by providing a more comprehensive and nuanced understanding of trends observed in the quantitative findings.

Third, there is a potential for recall bias among participants who were no longer actively staffing the service during the data collection period. In addition, the qualitative data analysis was conducted by a single coder and not independently in duplicate as per best practice. While this approach may introduce bias, the reflexive thematic approach used during analysis emphasizes the researcher’s interpretative engagement with the data over consensus or reliability measures [34]. This enables a richer, more cohesive analysis that upholds reflexivity and interpretative rigor. Nevertheless, future studies could enhance richer and deeper interpretations of the data by incorporating multiple coders in a collaborative and reflexive manner.

Furthermore, the clinical context during the data collection period was exclusively shaped by COVID-19, which presents unique challenges and care requirements compared with nonpandemic conditions typically managed in HaH programs. Despite this, our findings offer valuable insights into the use of telehealth tools, which remain relevant as HaH programs continue to expand and scale.

Finally, while the TUQ has demonstrated strong validity and reliability for assessing telehealth usability, it was originally developed for general telehealth users rather than providers. Nonetheless, we selected the TUQ for its comprehensiveness in evaluating core usability dimensions that are also relevant to health care providers. The comprehensive triangulation of data underscores the novelty of our study, as it demonstrates the adaptability of the TUQ beyond traditional end user settings and highlights its effectiveness in capturing the multidimensional experiences of health care providers engaging with telehealth within HaH.

### Conclusions

In summary, this study suggests that this generation of health care providers, though not considered to be formally trained in the use of telehealth tools, were generally adaptable and held positive perceptions regarding the usability of telehealth. Our findings further emphasize the equal importance of the health care providers as key stakeholders and end users (in addition to patients) in the development of telehealth tools. As HaH programs continue to scale internationally, further studies are needed to design and evaluate telehealth training programs for HaH healthcare providers and explore the influence of physical workspace design on health care provider effectiveness when using telehealth tools.

## Supplementary material

10.2196/56860 Multimedia Appendix 1 Good reporting of a mixed methods study criteria.

10.2196/56860 Multimedia Appendix 2 Detailed breakdown of Telehealth Usability Questionnaire scores.

10.2196/56860 Multimedia Appendix 3 Summary of subgroup analyses.

## Abbreviations

HaH: hospital-at-home
NUHS: National University Health System
TUQ: Telehealth Usability Questionnaire
