# Supplementary material for: Health Care Providers’ Experiences and Perceptions With Telehealth Tools in a Hospital-at-Home Program: Mixed Methods Study
Source: JMIR Hum Factors. 2025 Apr 17;12:e56860. doi: 10.2196/56860 (PMC12021374; doi:10.2196/56860)
Supplement: Multimedia Appendix 1 [file humanfactors-v12-e56860-s001.docx]

Table S1. Good Reporting of a Mixed Methods Study criteria

| **Guideline** | **Section: page** |
| --- | --- |
| Describe the justification for using a mixed methods approach to the research question | Methods – Study Design  Page 3 – 4 |
| Describe the design in terms of the purpose, priority and sequence of methods | Methods – Study Design  Page 3 – 4 |
| Describe each method in terms of sampling, data collection and analysis | Methods – Setting and Sample frame, Surveys, In-depth interviews, Data Analysis  Page 3 – 5 |
| Describe where integration has occurred, how it has occurred and who has participated in it | Methods – Data Analysis, Integrating quantitative and qualitative results  Page 12 and Table 3 |
| Describe any limitation of one method associated with the present of the other method | Discussion – Limitations  Page 16 |
| Describe any insights gained from mixing or integrating methods | Discussion  Page 15 – 16 |
