# Supplementary material for: Health Care Providers’ Experiences and Perceptions With Telehealth Tools in a Hospital-at-Home Program: Mixed Methods Study
Source: JMIR Hum Factors. 2025 Apr 17;12:e56860. doi: 10.2196/56860 (PMC12021374; doi:10.2196/56860)
Supplement: Multimedia Appendix 2 [file humanfactors-v12-e56860-s002.docx]

Table S2. TUQ responses for Teleconsultations (n=31)

| Item | | Mean (SD) | Median (IQR) | Range |
| --- | --- | --- | --- | --- |
| **Usefulness** | | 4.84 (0.30) | 5.0 (4.7–5.0) | 4.0 – 5.0 |
| 1. | Teleconsultations improve patients' access to healthcare services | 4.84 (0.37) | 5.0 (5.0–5.0) | 4.0 – 5.0 |
| 2. | Teleconsultations save travelling time to see patients in-person | 4.94 (0.25) | 5.0 (5.0–5.0) | 4.0 – 5.0 |
| 3. | Teleconsultations enabled me to provide a suitable level of care to my patients | 4.74 (0.44) | 5.0 (4.5–5.0) | 4.0 – 5.0 |
| **Ease of use** | | 4.72 (0.49) | 5.0 (4.7–5.0) | 3.2 – 5.0 |
| 4. | It was simple to conduct teleconsultations for my patients | 4.61 (0.67) | 5.0 (4.0–5.0) | 3.0 – 5.0 |
| 5. | It was easy to learn to use the teleconsultation systems | 4.84 (0.37) | 5.0 (5.0–5.0) | 4.0 – 5.0 |
| 6. | I believe my productivity in providing care has improved through teleconsultations | 4.65 (0.66) | 5.0 (4.5–5.0) | 3.0 – 5.0 |
| 7. | I feel comfortable interacting with the telehealth display systems | 4.74 (0.51) | 5.0 (5.0–5.0) | 3.0 – 5.0 |
| 8. | I enjoy using teleconsultations as part of delivering clinical care | 4.71 (0.59) | 5.0 (5.0–5.0) | 3.0 – 5.0 |
| 9. | The functions of teleconsultation systems are simple and easy to understand | 4.77 (0.56) | 5.0 (5.0–5.0) | 3.0 – 5.0 |
| **Effectiveness** | | 4.31 (0.61) | 4.4 (4.0–4.8) | 2.8 – 5.0 |
| 10. | Teleconsultations were able to perform the necessary functions I have requested for | 4.68 (0.54) | 5.0 (4.0–5.0) | 3.0 – 5.0 |
| 11. | I was able to easily talk to the patient using the teleconsultation system | 4.45 (0.77) | 5.0 (4.0–5.0) | 3.0 – 5.0 |
| 12. | I was able to easily hear the patient clearly using the teleconsultation system | 4.29 (0.74) | 4.0 (4.0–5.0) | 3.0 – 5.0 |
| 13. | I felt that I was able to express myself and communicate effectively through the teleconsultations | 4.48 (0.72) | 5.0 (4.0–5.0) | 2.0 – 5.0 |
| 14. | Seeing a patient online was similar to seeing a patient in-person | 3.65 (0.91) | 4.0 (3.0–4.0) | 2.0 – 5.0 |
| **Reliability** | | 4.06 (0.57) | 4.0 (3.8–4.3) | 3.0 – 5.0 |
| 15. | The experience of a teleconsultation is similar to that of an in-person consult | 3.74 (0.82) | 4.0 (3.0–4.0) | 2.0 – 5.0 |
| 16. | Whenever technical difficulties were encountered during teleconsultations, I was able to recover from them quickly | 4.26 (0.58) | 4.0 (4.0–5.0) | 3.0 – 5.0 |
| 17. | When technical difficulties were encountered, the teleconsultation system was able to identify them promptly with the appropriate solutions | 4.19 (0.79) | 4.0 (4.0–5.0) | 2.0 – 5.0 |
| **Satisfaction** | | 4.73 (0.43) | 5.0 (4.6–5.0) | 3.3 – 5.0 |
| 18. | I feel comfortable communicating with the patient through teleconsultations | 4.58 (0.50) | 5.0 (4.0–5.0) | 4.0 – 5.0 |
| 19. | teleconsultation is an acceptable way to deliver healthcare services to patients | 4.84 (0.45) | 5.0 (5.0–5.0) | 3.0 – 5.0 |
| 20. | I would consider using teleconsultation again in different settings | 4.74 (0.51) | 5.0 (5.0–5.0) | 3.0 – 5.0 |
| 21. | Overall, I am satisfied with the implementation of teleconsultations | 4.74 (0.51) | 5.0 (5.0–5.0) | 3.0 – 5.0 |
| **Total TUQ score** | | 4.55 (0.44) | 4.7 (4.5–4.8) | 3.4 – 5.0 |

Table S3. TUQ responses for Vital Signs Monitoring (n=34)

| Item | | Mean (SD) | Median (IQR) | Range |
| --- | --- | --- | --- | --- |
| **Usefulness** | | 4.80 (0.36) | 5.0 (4.7–5.0) | 3.7 – 5.0 |
| 1. | Vital signs monitoring improves patients' access to healthcare services | 4.79 (0.48) | 5.0 (5.0–5.0) | 3.0 – 5.0 |
| 2. | Vital signs monitoring saves travelling time to see patients in-person | 4.85 (0.36) | 5.0 (5.0–5.0) | 4.0 – 5.0 |
| 3. | Vital signs monitoring enabled me to provide a suitable level of care to my patients | 4.76 (0.55) | 5.0 (5.0–5.0) | 3.0 – 5.0 |
| **Ease of use** | | 4.70 (0.43) | 5.0 (4.5–5.0) | 3.5 – 5.0 |
| 4. | It was simple to utilise the vital signs monitoring systems | 4.59 (0.74) | 5.0 (4.0–5.0) | 2.0 – 5.0 |
| 5. | It was easy to learn to use the vital signs monitoring systems | 4.65 (0.60) | 5.0 (4.0–5.0) | 3.0 – 5.0 |
| 6. | I believe my productivity in providing care has improved through vital signs monitoring systems | 4.71 (0.58) | 5.0 (5.0–5.0) | 3.0 – 5.0 |
| 7. | I feel comfortable interacting with the vital signs monitoring display systems | 4.79 (0.41) | 5.0 (5.0–5.0) | 4.0 – 5.0 |
| 8. | I enjoy using vital signs monitoring systems as part of delivering clinical care | 4.74 (0.57) | 5.0 (5.0–5.0) | 3.0 – 5.0 |
| 9. | The functions of vital signs monitoring systems are simple and easy to understand | 4.71 (0.52) | 5.0 (4.3–5.0) | 3.0 – 5.0 |
| **Effectiveness** | | 4.29 (0.59) | 4.3 (3.8–4.8) | 3.0 – 5.0 |
| 10. | The vital signs monitoring system was able to perform the necessary functions I have requested for | 4.71 (0.52) | 5.0 (4.3–5.0) | 3.0 – 5.0 |
| 11. | I was able to easily talk to the patient using the vital signs monitoring system | 4.15 (0.82) | 4.0 (3.3–5.0) | 3.0 – 5.0 |
| 12. | I was able to easily hear the patient clearly using the vital signs monitoring system | 4.15 (0.86) | 4.0 (4.0–5.0) | 2.0 – 5.0 |
| 13. | I felt that I was able to express myself and communicate effectively using the vital signs monitoring system | 4.29 (0.76) | 4.0 (4.0–5.0) | 3.0 – 5.0 |
| 14. | Monitoring a patient's vital signs virtually was similar to doing so in-person | 4.15 (0.86) | 4.0 (4.0–5.0) | 2.0 – 5.0 |
| **Reliability** | | 3.98 (0.87) | 4.0 (3.3–4.7) | 2.0 – 5.0 |
| 15. | The experience of using vital signs monitoring is similar to that of monitoring in-person | 3.94 (0.92) | 4.0 (3.3–5.0) | 2.0 – 5.0 |
| 16. | Whenever technical difficulties were encountered during vital signs monitoring, I was able to recover from them quickly | 3.97 (0.94) | 4.0 (3.0–5.0) | 2.0 – 5.0 |
| 17. | When technical difficulties were encountered, the telehealth system was able to identify them promptly with the appropriate solutions | 4.03 (1.06) | 4.0 (3.3–5.0) | 1.0 – 5.0 |
| **Satisfaction** | | 4.75 (0.35) | 4.8 (4.8–5.0) | 3.8 – 5.0 |
| 18. | I feel comfortable communicating with the patient using vital signs monitoring systems | 4.59 (0.56) | 5.0 (4.0–5.0) | 3.0 – 5.0 |
| 19. | Vital signs monitoring is an acceptable method to help deliver healthcare services to patients | 4.82 (0.39) | 5.0 (5.0–5.0) | 4.0 – 5.0 |
| 20. | I would consider using vital signs monitoring systems again in different settings | 4.79 (0.48) | 5.0 (5.0–5.0) | 3.0 – 5.0 |
| 21. | Overall, I am satisfied with the implementation of vital signs monitoring | 4.79 (0.48) | 5.0 (5.0–5.0) | 3.0 – 5.0 |
| **Total TUQ score** | | 4.52 (0.42) | 4.6 (4.3–4.9) | 3.5 – 5.0 |
