# Supplementary material for: Health Care Providers’ Experiences and Perceptions With Telehealth Tools in a Hospital-at-Home Program: Mixed Methods Study
Source: JMIR Hum Factors. 2025 Apr 17;12:e56860. doi: 10.2196/56860 (PMC12021374; doi:10.2196/56860)
Supplement: Multimedia Appendix 3 [file humanfactors-v12-e56860-s003.docx]

Table S4. Sub-group comparison for Teleconsultation

|  | Profession | | | Years of clinical experience | | | Prior telehealth experience | | |
| --- | --- | --- | --- | --- | --- | --- | --- | --- | --- |
|  | Doctor (n=22)  Mean (SD) | Other healthcare providers (n=12)  Mean (SD) | p-value** | 0-7* years (n=19)  Mean (SD) | >7 years (n=15)  Mean (SD) | p-value** | Has telehealth experience (n=23)  Mean (SD) | No telehealth experience (n=11)  Mean (SD) | p-value** |
| Usefulness | 4.91 (0.23) | 4.67 (0.37) | **0.03145** | 4.81 (0.35) | 4.87 (0.22) | 0.96 | 4.87 (0.27) | 4.79 (0.34) | 0.4855 |
| Ease of Use | 4.87 (0.30) | 4.35 (0.67) | **0.04717** | 4.69 (0.57) | 4.77 (0.38) | 0.9627 | 4.75 (0.49) | 4.67 (0.52) | 0.7721 |
| Effectiveness | 4.45 (0.42) | 3.96 (0.85) | 0.1345 | 4.32 (0.59) | 4.29 (0.66) | 0.9355 | 4.30 (0.62) | 4.33 (0.60) | 0.9334 |
| Reliability | 4.18 (0.49) | 3.78 (0.67) | 0.09756 | 4.15 (0.63) | 3.95 (0.47) | 0.209 | 4.07 (0.56) | 4.06 (0.61) | 0.75 |
| Satisfaction | 4.83 (0.30) | 4.47 (0.61) | 0.1252 | 4.69 (0.52) | 4.77 (0.30) | 0.6224 | 4.80 (0.44) | 4.59 (0.41) | 0.06815 |
| Total TUQ | 4.67 (0.29) | 4.24 (0.59) | 0.1825 | 4.54 (0.49) | 4.55 (0.37) | 0.5599 | 4.57 (0.43) | 4.50 (0.46) | 0.709 |

*based on median calculated for years of clinical experience

**Mann-Whitney U test, *p* < 0.05

Table S5. Sub-group comparison for Vital Signs Monitoring

|  | Profession | | | Years of clinical experience | | | Prior telehealth experience | | |
| --- | --- | --- | --- | --- | --- | --- | --- | --- | --- |
|  | Doctor (n=22)  Mean (SD) | Other healthcare providers (n=12)  Mean (SD) | p-value** | 0-7* years (n=19)  Mean (SD) | >7 years (n=15)  Mean (SD) | p-value** | Has telehealth experience (n=23)  Mean (SD) | No telehealth experience (n=11)  Mean (SD) | p-value** |
| Usefulness | 4.82 (0.34) | 4.78 (0.41) | 0.7537 | 4.72 (0.43) | 4.91 (0.20) | 0.2031 | 4.87 (0.31) | 4.67 (0.42) | 0.1195 |
| Ease of Use | 4.73 (0.42) | 4.64 (0.47) | 0.6104 | 4.80 (0.38) | 4.57 (0.47) | 0.05209 | 4.75 (0.42) | 4.59 (0.47) | 0.2146 |
| Effectiveness | 4.38 (0.53) | 4.12 (0.67) | 0.2153 | 4.48 (0.57) | 4.04 (0.54) | **0.02062** | 4.31 (0.52) | 4.24 (0.74) | 0.8816 |
| Reliability | 4.15 (0.73) | 3.67 (1.04) | 0.1707 | 4.21 (0.80) | 3.69 (0.89) | 0.09147 | 4.12 (0.81) | 3.70 (0.95) | 0.1915 |
| Satisfaction | 4.82 (0.25) | 4.63 (0.47) | 0.3222 | 4.80 (0.36) | 4.68 (0.33) | 0.0729 | 4.79 (0.30) | 4.66 (0.44) | 0.4877 |
| Total TUQ | 4.59 (0.34) | 4.39 (0.53) | 0.3656 | 4.63 (0.43) | 4.39 (0.38) | **0.03509** | 4.58 (0.36) | 4.40 (0.52) | 0.3952 |

*based on median calculated for years of clinical experience

**Mann-Whitney U test, *p* < 0.05
